# Association between sociodemographic characteristics and level of knowledge about oral cancer among Mexican dental health professionals: a cross-sectional online survey

**DOI:** 10.1186/s12909-022-03952-0

**Published:** 2022-12-16

**Authors:** Ana Lilia Gijón-Soriano, Liliana Argueta-Figueroa, Yobana Pérez-Cervera, Alfonso Enrique Acevedo-Mascarúa, Norma Ivonne González-Arratia-López-Fuentes, Martha Adelina Torres-Muñoz, María de los Angeles Moyaho-Bernal, Rafael Torres-Rosas

**Affiliations:** 1 División de posgrado, Facultad de Odontología, Universidad Autónoma “Benito Juárez” de Oaxaca, Av. Universidad S/N, Ex-Hacienda 5 Señores, 68120 Oaxaca de Juárez, Mexico; 2 CONACyT – Facultad de Odontología, Universidad Autónoma “Benito Juárez” de Oaxaca, Av. Universidad S/N, Ex-Hacienda 5 Señores, 68120 Oaxaca de Juárez, Mexico; 3 Laboratorio de Inmunología asociado al Centro de Estudios en Ciencias de la Salud y la Enfermedad, División de Posgrado, Facultad de Odontología, Universidad Autónoma “Benito Juárez” de Oaxaca, Av. Universidad S/N, Ex-Hacienda 5 Señores, 68120 Oaxaca de Juárez, Mexico; 4 Facultad de Ciencias de la Conducta Universidad Autónoma del Estado de México, Filiberto Gómez s/n, Barrio de Tlacopa, 50010 Toluca de Lerdo, Mexico; 5 Facultad de Estomatología, Benemérita Universidad Autónoma de Puebla, calle 31 Poniente #1304, Col. Los Volcanes, C. P. 72410 Puebla, Mexico

**Keywords:** Medicine, Dentistry, Health education

## Abstract

**Background:**

A good level of knowledge in dentists is crucial for an early diagnosis of oral cancer (OC). In Latin America there are a few studies of OC knowledge among dentist, those has been performed in Brazil, Colombia, and Chile, and their results showed low level of OC knowledge. On the other hand, there is no publication in which the level of knowledge of dentists in Mexico has been addressed. Therefore, this study aimed to assess knowledge of OC and to determine the association of the level of knowledge with sociodemographic characteristics among dentists in Mexico.

**Methods:**

A cross-sectional online survey was designed to obtain information via questionnaire. The questionnaire was developed in the Spanish language, and the content validity was determined. The study was conducted among Mexican dentists with a 23-item questionnaire that was designed to be anonymous. The sample size was calculated using the finite population formula. Based on the responses, the level of knowledge of OC was categorized as very low, low, regular, good, or excellent. Additionally, the association between sociodemographic characteristics and the level of knowledge about OC was evaluated.

**Results:**

This research was conducted on a sample of 387 dentists. Most of the respondents were general dentists and worked in urban zones. The majority of dentists lacked a specialty (76.7%). Additionally, most of the respondents were students (44.2%). The level of knowledge of the participants was between regular and good (77.8%). On the other hand, concerning self-evaluation, most of the participants considered their knowledge of OC to be regular (50.6%). In addition, there was no association between sociodemographic characteristics and knowledge about OC.

**Conclusions:**

This research identified some weaknesses in most Mexican dentists’ knowledge of OC.

**Supplementary Information:**

The online version contains supplementary material available at 10.1186/s12909-022-03952-0.

## Background

In 2008, two-thirds of all deaths globally were caused by chronic noncommunicable diseases, mainly cardiovascular diseases (48%) and cancer (21%). Later, in 2016, noncommunicable diseases were responsible for 71% of the 57 million deaths that occurred worldwide, including mainly cardiovascular diseases (44%), cancers (9%), chronic respiratory diseases (9%), and diabetes (4%) [1]. Nevertheless, the evidence shows that cancer mortality decreased by 12% between 2008 and 2016. However, a recurrent observation is the displacement of cancers related to infectious diseases and poverty by cancers linked to lifestyle [2]. In addition, exposure to extreme environmental conditions, behavioral risk factors, and lack of knowledge are indicators of the wide variation in the global incidence [3].

Oral cancer (OC) is the sixth most common malignant neoplasm in the world. It has an annual incidence of more than 300,000 cases. The most predominant OC is oral squamous cell carcinoma (OSCC). Oral potentially malignant disorders (OPMDs), such as lichen planus, leukoplakia, erythroplakia, inflammatory oral submucosa, oral submucous fibrosis, oral lupus erythematosus, and some hereditary conditions, are indicators of the preclinical phase of OC [4]. Five years after diagnosis, the overall survival rate is approximately 62% for cancer of the lip, tongue, gums, and other parts of the mouth, including the floor of the mouth, salivary glands, tonsils, and oropharynx [5]. One obstacle for timely OC treatment involves the early diagnosis of the disease. Most OCs are diagnosed at later stages, mainly because, in the early stages, many patients are asymptomatic and do not seek medical consultation until they notice evident signs, such as mass or significant deformations in the mouth or neck, or symptoms such as pain or bleeding [6]. Generally, the patient delays the diagnosis because he or she is not aware of the importance of seeking a diagnostic dental appointment after 15 days without a lesion healing; nevertheless, delay can also result from an incorrect dentistry approach, with the dentist not suspecting oral malignancy and not diagnosing it promptly and adequately [7].

An underestimation of the worldwide prevalence of OC is very likely. In fact, there are 270,000 new cases and 145,000 deaths annually; of those, two-thirds occur in developing countries such as México. One of the main reasons is that low- and middle-income countries have limited health resources focused on early cancer diagnosis [8, 9]. The clinical and pathological stage at diagnosis is the most critical conditional factor for prognosis. The early detection of OPMDs and oral malignant lesions by the dentist results in an early referral to a specialist, appropriate intervention, and, consequently, better prognosis and survival rates and fewer sequelae from treatment.

There are several OC knowledge studies among dentists in diverse countries around the world. For instance, Wimamardhani, et al. performed a study in Indonesia to assess the OC knowledge level in dentists, and they reported that the dentists had a considerable OC knowledge level of the main risk factors, although some gaps in the diagnostic procedures [10]. Ahmed and Naidoo reported a good level of identification of oral lesions (non-healing ulcers and white or red patches) and early lesions of OC among dentists in Khartoum, Sudan [11]. On the other hand, Khattab et al. reported that the overall OC knowledge level in upper Egypt was 31.8% [12], Golburean, et al. in a multi-country study (Moldova, Belarus, and Armenia), reported a knowledge score of 7.5 ± 2.7 (range = 0–14, representing 0–8 as low level) [13].

In Latin America, OC knowledge among dentists from Brazil, Colombia, and Chile was studied. Leonel, et al. reported low OC knowledge and low confidence level required for diagnosing OC in dentists from Brazil [14]. Also, Rocha-Buelvas, et al. reported that around 50% of the respondents to their questionnaire about OC knowledge failed to answer in a survey in Colombia. Furthermore, Stillfried, et al. reported similar findings in Chile [15]. A low level of knowledge leads to inadequate diagnosis, and dentist OC knowledge is crucial to understanding screening programs’ feasibility and effectiveness. As far as we know, there is no publication in which the knowledge level of dentists in Mexico has been addressed. Therefore, this research aimed to determine dentists’ knowledge level about OC in Mexico.

## Methods

### Study design and questionnaire

A cross-sectional online survey was performed to obtain the data from Mexican dentists following the “NORMA Oficial Mexicana NOM-012-SSA3-2012 Que establece los criterios para la ejecución de proyectos de investigación para la salud en seres humanos” [16]. The study was conducted with 387 Mexican dentists who voluntarily answered a questionnaire designed to be anonymous between February 2021 and July 2021. The data obtained in this study were kept confidential. Ethical approval (ref no: EO20210001FO) was obtained from the local Human Research Ethics Committee (Comité de ética en investigación de la Facultad de Odontología, Universidad Autónoma “Benito Juárez” de Oaxaca”). Also, informed consent was obtained from each participant after clarification of the study characteristics and objectives.

An expert educational team that included two dentists and one medical doctor formulated the questions for assessing oral knowledge of cancer. After that, the content validity of the OC questionnaire was carried out with an expert panel that included six professors with the specialties of oral pathology (*n* = 2), maxillofacial surgery (*n* = 2), and health education (*n* = 2). This expert team also helped to judge the content validity of the items initially selected for the questionnaire, and the content validity coefficient (Cvc) was calculated for the content validity. A total content validity coefficient (CVCt) ≥0.80 was considered satisfactory [17–19]. After the first measurement, the questionnaire was modified according to the expert panel’s recommendations to obtain an adequate coefficient, as shown in Table 1. The questionnaire was developed in the Spanish language.

**Table 1:** Results of the questionnaire content validation

Question	SX	Mx	Cvci	Pei	CVCt	SX	Mx	Cvci	Pei	Cvct
	First measurement					Second measurement				
1	58	2.32	0.77	0.04	0.74	63	2.52	0.84	0.04	0.80
2	64	2.56	0.85	0.04	0.82	64	2.56	0.85	0.04	0.82
3	69	2.76	0.92	0.04	0.88	69	2.76	0.92	0.04	0.88
4	68	2.72	0.91	0.04	0.87	69	2.76	0.92	0.04	0.88
5	65	2.60	0.87	0.04	0.83	65	2.60	0.87	0.04	0.83
6	68	2.72	0.91	0.04	0.87	70	2.80	0.93	0.04	0.90
7	69	2.76	0.92	0.04	0.88	69	2.76	0.92	0.04	0.88
8	70	2.80	0.93	0.04	0.90	70	2.80	0.93	0.04	0.90
9	70	2.80	0.93	0.04	0.90	70	2.80	0.93	0.04	0.90
10	46	1.84	0.61	0.04	0.58	64	2.56	0.85	0.04	0.82
11	69	2.76	0.92	0.04	0.88	69	2.76	0.92	0.04	0.88
12	60	2.40	0.80	0.04	0.76	63	2.52	0.84	0.04	0.80
13	46	1.84	0.61	0.04	0.58	66	2.64	0.88	0.04	0.84
14	60	2.40	0.80	0.04	0.76	67	2.68	0.89	0.04	0.86

*Sx* Sum of the scores assigned by each judge, *Mx* Maximum value, *CVCi* Item content validity coefficient, *Pei* probability of failure, *CVCt* Total content validity coefficient

The final version of the questionnaire was established with the items related to knowledge of OC from the content validity assessment by a second evaluation with the Cvc. The final questionnaire comprised close-ended questions, with 3 domains of 23 questions: were sociodemographics, knowledge of OC, and self-evaluation. The domains and characteristics considered in this study are shown in Table 2, and the questionnaire of the domain knowledge of OC is shown in Table S1.

**Table 2:** Domains assessed in the questionnaire of levels of knowledge about oral cancer of dentists

Domain	# Items	Measurement	Response Choice
Demography	9	Socio-demographic characteristics: Gender, Age, Region of the University (bachelor’s degree completion), Region of residence, Location of practice, Specialty, Academic degree, Occupation, Range of years after bachelor’s degree completion.	Multiple choice.
Level of Knowledge	13	Oral potentially malignant disorders recognition by photography, general knowledge, risk factors, and diagnosis of oral cancer.	Multiple choice
Self-evaluation	1	Self-evaluation	Multiple choice

There were 4 items on the recognition of OPMDs or suggestive OC in photographs, 4 items on the knowledge of the clinical aspects of OC and 5 items on the knowledge of epidemiology and risk factors for OC (Table S1). The total score ranged from 0 to 13 points. The participants received 1 point for each correct response and were categorized based on the number of correct answers (level of knowledge: 0–2 very low, 3–5 low, 8–6 regular, 9–11 good, 12–13 excellent). Additionally, the self-evaluation was categorized as very low, low, regular, good, and excellent.

### Sample

The sample size for the final questionnaire application was calculated using the finite population formula considering 115,000 dentists from Mexico [20] as the population, a 95% confidence level, a standard deviation of 0.5, and a margin of error (confidence interval) of 5%. The calculation resulted in a sample of 383 participants.

### Statistical analysis

The Cvc was calculated using the Excel program (Microsoft office 365). Descriptive analysis, Cramer’s V, and Spearman’s correlation test were calculated using the IBM SPSS Statistics 21 program (IBM Corp.). The value of *p* was considered statistically significant if *p* ≤ 0.05.

## Results

This research was conducted on a sample of 387 dentists, including 276 women (71.3%) and 111 men (28.7%). The mean age of the respondents was 29.34 ± 11.270 (range = 18–68) years old. The respondents completed their bachelor’s degrees and resided in different regions of México, mainly the Southwest. Most of the respondents were general dentists and worked in urban zones. The majority of dentists lacked a specialty (76.7%). Additionally, most of the respondents were students (44.2%), followed by those with bachelor’s degree (33.1%). Finally, the dentists’ main place of occupation was in a dental office (49.4%) (Table 3).

**Table 3:** Socio-demographic characteristics of participants

Variable	Answer	N	%
Sex	Female	276	71.3
	Male	111	28.7
Region of the University (bachelor’s degree completion)	Central	48	12.4
	West	23	5.9
	Southwest	303	78.3
	Northwest	2	0.5
	Northeast	4	1.0
	Southeast	7	1.8
Region of residence	No answer	18	4.7
	Central	39	10.1
	West	20	5.2
	Southwest	298	77.0
	Northwest	4	1.0
	Northeast	2	0.5
	Southeast	6	1.6
Location of practice	No answer	18	4.7
	Urban zone	196	50.6
	Rural zone	173	44.7
Specialty	Without Specialty	297	76.7
	Orthodontist	30	7.8
	Prosthodontist	15	3.9
	Endodontist	23	5.9
	Pediatric dentist	15	3.9
	Periodontist	3	0.8
	Oral surgeon	4	1.0
Academic degree	Student	171	44.2
	Bachelor’s degree	128	33.1
	Specialty	40	10.3
	Master	46	11.9
	Doctorate	2	0.5
Occupation	Student	171	44.2
	Dental office	191	49.4
	Teacher	6	1.6
	Researcher	1	0.3
	Administrative	3	0.8
	Unemployed	15	3.9
Range of years after bachelor’s degree completion	Student	189	48.8
	1–10 years	109	28.2
	11–20 years	45	11.6
	> 20 years	44	11.4

### Knowledge level of participants

We evaluated whether the dentists recognized images related to the clinical impression of lesions. 59.4% of dentist recognized a hematoma, 94.1% of respondents recognized an oral carcinoma image, 68.2% of respondents recognized the verrucous carcinoma image, and 78.6% of the dentists recognized the images of OPMDs fibrous hyperplasia.

In addition, the majority of dentists knew clinical aspects of OC such as the most common feature in patients with initial OC (69%), the main characteristics of the cervical lymph nodes in a patient with OC and metastasis (49.6%), the gold standard study for OC diagnosis (82.2%), and the main structures in the palpation during the physical examination (89.4%). Concerning epidemiological aspects, the majority of the dentists recognized the most frequent anatomical region of OC presentation (64.3%) and the most common type of OC (70.8%); however, most of the dentists did know the age at which there is a higher prevalence (39.0%), the risk factors (22.0%), and the preventive habits in the field of OC (33.1%).

Ultimately, the mean total score was 8.20 ± 2.364 (range = 2–13) and the knowledge level of the participants was between regular and good (77.8%), as shown in Table 4. On the other hand, concerning self-evaluation, most of the participants considered their knowledge of OC to be regular (50.6%), followed by low, good, and very low (31.8, 10.1, and 7.2%, respectively.

**Table 4:** Percentage of correct and incorrect answers and knowledge level from participants

# Question	Answer	N	%
Q1	Wrong	157	40.6
	Right	230	59.4
Q2	Wrong	23	5.9
	Right	364	94.1
Q3	Wrong	123	31.8
	Right	264	68.2
Q4	Wrong	83	21.4
	Right	304	78.6
Q5	Wrong	120	31.0
	Right	267	69.0
Q6	Wrong	138	35.7
	Right	249	64.3
Q7	Wrong	113	29.2
	Right	274	70.8
Q8	Wrong	236	61.0
	Right	151	39.0
Q9	Wrong	195	50.4
	Right	192	49.6
Q10	Wrong	69	17.8
	Right	318	82.2
Q11	Wrong	302	78.0
	Right	85	22.0
Q12	Wrong	41	10.6
	Right	346	89.4
Q13	Wrong	259	66.9
	Right	128	33.1
Level of knowledge	Very low	4	1.0
	Low	44	11.4
	Regular	170	43.9
	Good	131	33.9
	Excellent	38	9.8
	Total	387	100.0

### Association between sociodemographic characteristics and the knowledge level about OC

Approximately half of the dentists had between good and excellent knowledge about OC without differences between sexes (female = 44.20%, male = 42.34%). Additionally, there were no differences concerning the region of university (*p* > 0.5), region of residence (*p* > 0.5) or location of practice (*p* > 0.5) with the knowledge about OC. Nevertheless, there was no association between the knowledge level and the previous sociodemographic characteristics (Table 5).

**Table 5:** Results of the association of the variables studied and the knowledge level about oral cancer of the participants

			VL	L	R	G	E	Total
Sex	Female	Count	3	32	119	101	21	276
		%	75.0%	72.7%	70.0%	77.1%	55.3%	71.3%
	Male	Count	1	12	51	30	17	111
		%	25.0%	27.3%	30.0%	22.9%	44.7%	28.7%
							Coefficient	*P*
				**Cramér’s V**			0.136	0.129
ROU	Central	Count	0	3	24	17	4	48
		%	0.0%	6.8%	14.1%	13.0%	10.5%	12.4%
	West	Count	1	3	8	10	1	23
		%	25.0%	6.8%	4.7%	7.6%	2.6%	5.9%
	Southwest	Count	3	34	134	99	33	303
		%	75.0%	77.3%	78.8%	75.6%	86.8%	78.3%
	Northwest	Count	0	0	1	1	0	2
		%	0.0%	0.0%	0.6%	0.8%	0.0%	0.5%
	Northeast	Count	0	0	2	2	0	4
		%	0.0%	0.0%	1.2%	1.5%	0.0%	1.0%
	Southeast	Count	0	4	1	2	0	7
		%	0.0%	9.1%	0.6%	1.5%	0.0%	1.8%
							Coefficient	*P*
				**Cramér’s V**			0.124	0.248
ROR	No answer	Count	0	3	7	5	3	18
		%	0.0%	6.8%	4.1%	3.8%	7.9%	4.7%
	Central	Count	0	4	20	11	4	39
		%	0.0%	9.1%	11.8%	8.4%	10.5%	10.1%
	West	Count	0	3	7	9	1	20
		%	0.0%	6.8%	4.1%	6.9%	2.6%	5.2%
	Southwest	Count	4	31	135	98	30	298
		%	100.0%	70.5%	79.4%	74.8%	78.9%	77.0%
	Northwest	Count	0	0	1	3	0	4
		%	0.0%	0.0%	0.6%	2.3%	0.0%	1.0%
	Northeast	Count	0	0	0	2	0	2
		%	0.0%	0.0%	0.0%	1.5%	0.0%	0.5%
	Southeast	Count	0	3	0	3	0	6
		%	0.0%	6.8%	0.0%	2.3%	0.0%	1.6%
							Coefficient	*P*
				**Cramér’s V**			0.126	0.428
LOP	No answer	Count	0	3	7	5	3	18
		%	0.0%	6.8%	4.1%	3.8%	7.9%	4.7%
	Urban	Count	3	20	85	74	14	196
		%	75.0%	45.5%	50.0%	56.5%	36.8%	50.6%
	Rural	Count	1	21	78	52	21	173
		%	25.0%	47.7%	45.9%	39.7%	55.3%	44.7%
							Coefficient	*P*
				**Cramér’s V**			0.095	0.537

*ROU* Region of the university, *ROR* Region of residence, *LOP* Location of practice, (Very low), *L* Low, *R* Regular, G Good, *E* Excellent

There were no associations between academic degree, specialty, or occupation and knowledge about OC (Table 6).

**Table 6:** Results of comparison of the variables studied and the knowledge level about oral cancer of the participants

			VL	L	R	G	E	Total
Specialty	Without	Count	4	36	124	100	33	297
	Specialty	%	1.3%	12.1%	41.8%	33.7%	11.1%	100.0%
	Orthodontist	Count	0	3	15	11	1	30
		%	0.0%	10.0%	50.0%	36.7%	3.3%	100.0%
	Prosthodontist	Count	0	0	10	4	1	15
		%	0.0%	0.0%	66.7%	26.7%	6.7%	100.0%
	Endodontist	Count	0	3	13	7	0	23
		%	0.0%	13.0%	56.5%	30.4%	0.0%	100.0%
	Pediatric	Count	0	2	6	6	1	15
	Dentist	%	0.0%	13.3%	40.0%	40.0%	6.7%	100.0%
	Periodontist	Count	0	0	1	1	1	3
		%	0.0%	0.0%	33.3%	33.3%	33.3%	100.0%
	Oral surgeon	Count	0	0	1	2	1	4
		%	0.0%	0.0%	25.0%	50.0%	25.0%	100.0%
							Coefficient	*P*
				**Cramér’s V**			0.100	0.906
AD	Student	Count	2	22	66	54	27	171
		%	50.0%	50.0%	38.8%	41.2%	71.1%	44.2%
	Bachelor’s	Count	2	14	60	47	5	128
	Degree	%	50.0%	31.8%	35.3%	35.9%	13.2%	33.1%
	Specialty	Count	0	4	18	17	1	40
		%	0.0%	9.1%	10.6%	13.0%	2.6%	10.3%
	Master	Count	0	4	25	12	5	46
		%	0.0%	9.1%	14.7%	9.2%	13.2%	11.9%
	xam	Count	0	0	1	1	0	2
		%	0.0%	0.0%	0.6%	0.8%	0.0%	0.5%
							Coefficient	*P*
				**Spearman’s Correlation**			−0.060	0.236
Ocupation	Student	Count	3	21	66	55	26	171
	Dental	%	75.0%	47.7%	38.8%	42.0%	68.4%	44.2%
		Count	1	20	90	70	10	191
	Office	%	25.0%	45.5%	52.9%	53.4%	26.3%	49.4%
	Teacher	Count	0	0	3	3	0	6
		%	0.0%	0.0%	1.8%	2.3%	0.0%	1.6%
	Researcher	Count	0	0	1	0	0	1
		%	0.0%	0.0%	0.6%	0.0%	0.0%	0.3%
	Administrative	Count	0	0	3	0	0	3
		%	0.0%	0.0%	1.8%	0.0%	0.0%	0.8%
	Unemployed	Count	0	3	7	3	2	15
		%	0.0%	6.8%	4.1%	2.3%	5.3%	3.9%
							Coefficient	*P*
				**Cramér’s V**			0.119	0.339
RYABDC	Student	Count	4	25	74	56	30	189
		%	100.0%	56.8%	43.5%	42.7%	78.9%	48.8%
	1–10 years	Count	0	9	51	46	3	109
		%	0.0%	20.5%	30.0%	35.1%	7.9%	28.2%
	11–20 years	Count	0	6	21	15	3	45
		%	0.0%	13.6%	12.4%	11.5%	7.9%	11.6%
	> 20 years	Count	0	4	24	14	2	44
		%	0.0%	9.1%	14.1%	10.7%	5.3%	11.4%
							Coefficient	*P*
				**Spearman’s Correlation**			−0.054	0.011

*AD* Academic degree, *RYABDC* range of years after bachelor’s degree completion, *VL* Very low, *L* Low, *R* Regular, *G* Good, *E* Excellent

The knowledge level was not associated with age, range of years after bachelor’s degree completion, or academic degree. Additionally, the self-evaluation of knowledge had no association with age, range of years after bachelor’s degree completion, or academic degree (Table 7).

**Table 7:** Correlation between socio-demographic ordinal variables and total punctuation, knowledge level and self-evaluation of knowledge

Spearman’s Rank Correlation Coefficient		Total punctuation	Level of knowledge	Self-evaluation of knowledge
Age	Coefficient	−0.073	−0.073	− 0.076
	*p* value	0.150	0.154	0.135
RYABDC	Coefficient	−0.051	− 0.054	−0.072
	*p* value	0.320	0.286	0.159
AD	Coefficient	−0.050	−0.060	−0.027
	*p* value	0.327	0.236	0.594
Total punctuation	Coefficient	NA	NA	0.319
	*p* value	NA	NA	0.0001
Level of knowledge	Coefficient	NA	NA	0.329
	*p* value	NA	NA	0.0001

*RYABDC* Range of years after bachelor’s degree completion), *AD* Academic degree, *NA* No applicable

## Discussion

OC mortality could be significantly reduced in the population through early diagnosis. There are well known OC risk factors, epidemiological aspects, clinical indicators, and OPMDs. Therefore, we directed our questionnaire to those aspects of OC to determine dentists’ knowledge.

Different research groups have assessed delayed diagnosis of OC. The diagnostic delay can be categorized into a) patient delay, b) professional delay, and c) overall diagnostic delay. Professional delay concerns the period between the patient’s first consultation with the professional and the definitive pathological diagnosis. Although there is no agreement concerning whether patient delay and professional delay is the prevalent cause of failure in early diagnosis, both factors must be considered [21]. Ultimately, the quality and safety of a diagnosis are determined by the competencies that health professionals and patients bring to the diagnostic process [22]. Likewise, there is a high level of concern about health education and consciousness in the general public. A survey on the levels of public awareness about OC revealed that only 65.4% of the respondents had heard of OC, and 23.8% indicated doctors as a source of information about OC; this research showed unsatisfactory levels of patient awareness of the risk factors and symptoms of OC [23]. In the present work, the Cvc allowed for the quantitative measurement and evaluation of the content validity of a given data collection instrument (questionnaire) on a scale of 0 to 1 using the expert technique. Although the kappa coefficient has been used for validation in other research, this coefficient only measures the agreement between judges. However, concordance is a necessary but not sufficient condition to ensure the content validity of a questionnaire. Therefore, the Cvc measures the validity and concordance not only of the total questionnaire (Cvct) but also of each item (Cvci) to facilitate the questionnaire construction process [19].

Different research groups have studied dentists’ knowledge regarding OC and its early detection and have pointed out that it is fundamental to enhance knowledge about OC [12, 24–28]. However, to the best of our knowledge, there are no studies aiming to determine the level of OC knowledge of Mexican dental health professionals. In this study, only approximately half of the dentists had good or excellent knowledge about OC; these results are similar to those of other studies performed among identical participants [12, 26].

Several risk factors for OC have been described, such as chemical factors from tobacco or alcohol consumption, biological factors such as human papillomavirus, and dietary deficiencies [29]. In this study, the results showed a deficient knowledge of risk factors for OC (Q11); these results may be related to the poor knowledge concerning the low consumption of fruits and vegetables as a risk factor for OC, which agrees with the results of Jboor et al. [30]. Additionally, we found that most dentists had good knowledge of the clinical presentation of OPMDs and OC. This result was consistent with recent studies that showed that dentists were conscious of the clinical presentation of OPMDs and OC [26, 30].

The most common OC is OSCC; this cancer is more prevalent in men over 45 years old from low- and middle-income countries. OSCC includes extraoral (lip cancer, the primary type of OSCC) and intra-OC, mainly found in the tongue [31]. In this study, a high percentage of the participants recognized squamous cell carcinoma as the most common form of OC; this result was consistent with recent studies [30].

Traditionally, it has been considered that the scores obtained in knowledge evaluations through a structured written exam with multiple choice answers follow a normal distribution. However, it is currently considered that test scores with polytomous items do not fit the normal curve. Such scores have been shown to be asymmetrical [32]. The mean of the curve tends to the right side, and more values are found in the upper tail, so the curve is asymmetric.

Many reliability index measures have been used to prove the test reliability, including Cronbach’s alpha, Spearman’s rank correlation, and R^2^ coefficient determinants. All these indexes have been used because no single tool has been considered precise enough or adequate. A study stated that Cronbach’s alpha coefficient is not sufficient for measuring the reliability of a test [33]. Cronbach’s alpha was developed to measure the consistency of the content across items in psychology. Although it is considered a good index, the measure is affected by the number of items and the number of participants. In addition, a high coefficient could reflect redundancy instead of reliability when several items ask the same questions in different ways. Additionally, the Cronbach’s alpha test is an inappropriate test if the item does not follow a pattern of responses, such as when there is a correct answer and the other answers are wrong [34]. Therefore, Cronbach’s alpha was not used to determine the reliability of our questionnaire.

In the present study, a probabilistic sample was not obtained since all the respondents who wished to participate were enrolled. Although the invitation to participate was made in various ways, trying to have enough participation from Mexican dentists, we did not have much participation, so the goal was to reach the necessary number of participants according to the sample calculation. A sample with *n* > 200 can provide reasonably accurate parameter estimation in the case of a test with up to 15 items [35]. Therefore, the sample size of this study was adequate. However, one of the limitations of this study is that there was not a homogeneous number of study participants per region. In addition, the lack of participation of graduated dentists was in part due to a certain fear of taking knowledge tests. Because of this, before answering the questionnaire on the digital platform, the participants were assured that the results of each individual evaluation would be confidential [36]. In addition, when sending the questionnaire, each participant obtained her or his total score with the wrong answers and the correct answer for each answer. Additionally, to avoid bias, it was not possible to correct the responses after submitting the questionnaire or to submit the questionnaire more than once by the same person to avoid duplicate responses [37]. On the other hand, we expected to find an important difference in the knowledge of oral pathologists and maxillofacial surgeons, as other studies have been reported [38]; however, in Mexico, there are very few professionals in these specialties compared to others, and this was reflected in the sample, which was an important limitation of this study.

Various legal systems regulate the professional practice of dentistry in Mexico, the main ones being in article 5° of the Political Constitution of the United Mexican States and article 79° of the General Health Law [39, 40]. Patients need to have highly professional services to exercise their right to health. Due to this, there have been various efforts to achieve certification in the area of dentistry, which have been implemented through the Mexican Dental Association (Asociación Dental Mexicana) and the National Federation of Colleges of Dental Surgeons (Federación Nacional de Colegios de Cirujanos Dentistas, S.A. de C.V). These nongovernmental organizations are responsible for certification in dentistry. However, this certification is voluntary.

The professional certification for dentists in Mexico is not mandatory because national laws consider that the professional title, which is legally issued and registered by the competent educational authorities, is sufficient to demonstrate that dentists have the necessary knowledge to exercise their profession. However, according to the human right to health, the patient should receive highly professional services, and the central element of health service quality is a professional update verified through certification. This is exemplified by the fact that certification can offer several benefits for dentists, employers, academic institutions, certification associations, and dental patients. The patients are served by certification, as it creates a standard for professionals and provides assurance that the certification associations have met rigorous requirements in areas of practice. Additionally, certification can benefit certification associations by validating knowledge, identifying professional achievement, evaluating knowledge weaknesses, and promoting professional credibility [41].

In the last 50 years, dental education has been transformed, and evaluation instruments are necessary to prevent the deterioration of dental education in Mexico; therefore, this culture of evaluation, accreditation, and certification is no longer only a requirement of globalization but also a need of the present times, which requires qualified dentistry professionals to solve the oral health problems of the population. In that sense, the dentist’s certification in Mexico should be mandatory, as is the case for medical doctors.

## Conclusions

This research identified some weaknesses in most dentists’ knowledge of OC, the knowledge level of the participants was between regular and good without associations between knowledge level of OC and sociodemographic characteristics.

## Supplementary Information

**Additional file 1: Table S1.** Domain knowledge’s questionnaire; **Fig. S1.** Hematoma image, **Fig. S2.** Oral carcinoma image, **Fig. S3.** Verrucous carcinoma image, **Fig. S4.** Oral fibrous hyperplasia image.

## Data Availability

The data that support the findings of this study are available from the corresponding author upon reasonable request.
