# Supplementary material for: Association between sociodemographic characteristics and level of knowledge about oral cancer among Mexican dental health professionals: a cross-sectional online survey
Source: BMC Med Educ. 2022 Dec 16;22:874. doi: 10.1186/s12909-022-03952-0 (PMC9756605; doi:10.1186/s12909-022-03952-0)
Supplement: Supplementary file 1 — Additional file 1: Table S1. Domain knowledge’s questionnaire; Fig. S1. Hematoma image, Fig. S2. Oral carcinoma image, Fig. S3. Verrucous carcinoma image, Fig. S4. Oral fibrous hyperplasia image. [file 12909_2022_3952_MOESM1_ESM.docx]

| Table S1: Domain knowledge's questionnaire | |
| --- | --- |
| Q1.- Which is the presumptive lesion inside the green box in photograph #1? | |
| Ulcer | Figure S1: Hematoma image |
| Papule |  |
| Vesicle |  |
| Macular lesion |  |
| Hematoma |  |
| Q2.-What is the presumptive diagnosis based on the lesion located inside the green box in photograph #2? | |
| Lichen planus | Figure S2: Oral carcinoma image |
| Candidiasis |  |
| Oral carcinoma |  |
| Cheilitis |  |
| White sponge nevus |  |
| Q3.- What is the presumptive diagnosis based on the lesion located inside the green box in photograph #3? | |
| Verrucous carcinoma | Figure S3: Verrucous carcinoma image |
| Behcet's síndrome |  |
| Lichen planus |  |
| Cheilitis |  |
| Wegener's granulomatosis |  |
| Q4.- What is the presumptive diagnosis based on the lesion located inside the green box in photograph #4? | |
| Verrucous carcinoma  Behcet's syndrome  Fibrous hyperplasia  Cheilitis  Wegener's granulomatosis | Figure S4: Oral fibrous hyperplasia image |
| Q5.- What is the most common feature in patients with initial oral cancer? | |
| Profuse salivation |  |
| Painless ulcer |  |
| Hard nodule |  |
| Intense pain |  |
| Cryptogenic fever |  |
| Q6.- What is the most frequent anatomical region of oral cancer presentation? | |
| Tongue |  |
| Palate |  |
| Maxillary bones |  |
| Superior lip |  |
| Salivary glands |  |
| Q7.- What is the most common type of oral cancer? | |
| Lymphoma |  |
| Squamous cell carcinoma |  |
| Kaposi's sarcoma |  |
| Salivary gland adenoma |  |
| Verrucous carcinoma |  |
| Q8.- What is the age group with the higher prevalence of oral cancer? | |
| < 5 years |  |
| 6-14 years |  |
| 15-24 years |  |
| 25-60 years |  |
| ≥ 61 years |  |
| Q9.- What are the main characteristics of the cervical lymph nodes in a patient with oral cancer and metastasis? | |
| Hard, painful and movable |  |
| Hard, painless, and movable or fixed |  |
| Soft, painful, and movable |  |
| Soft, painless, and movable or fixed |  |
| Soft, painful, fixed |  |
| Q10.- What is the gold standard study for oral cancer diagnosis? | |
| Flow cytometry |  |
| Pipecolinic acid quantification |  |
| VELscope |  |
| Orascoptic DK |  |
| Histopathological study of oral biopsy |  |
| Q11.- All the following are risk factors for oral cancer, EXCEPT: | |
| Previous diagnoses of another cancer | |
| Low consumption of fruits and vegetables |  |
| Unprotected oral sex |  |
| Tooth infection |  |
| Smoking |  |
| Q12.- Which elements must the dentist palp during the physical examination of oral cancer? | |
| Preauricular, retroauricular, and occipital ganglion chains | |
| Sinuses |  |
| Endoperiodontal lesion |  |
| Periodontal probing |  |
| Maxillary sinuses |  |
| Q13.- Which of the following are preventive habits against oral cancer? | |
| Electronic cigarette consumption  Daily flossing  Use of mouthwashes with benzydamide  Fruit and vegetable consumption  Tooth brushing 3 times a day |  |


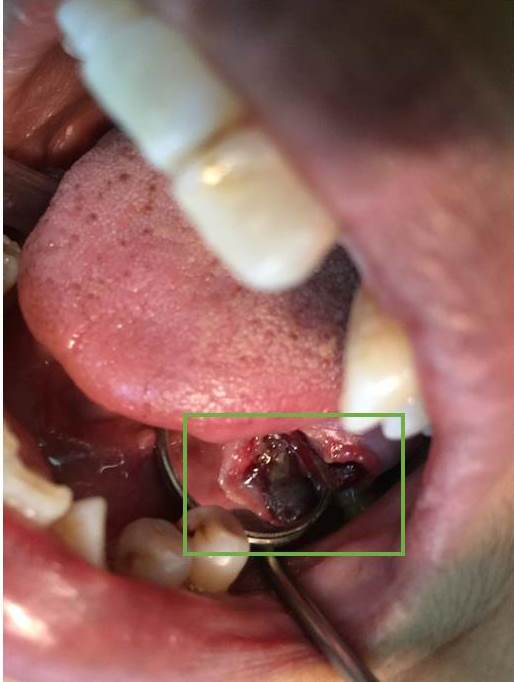

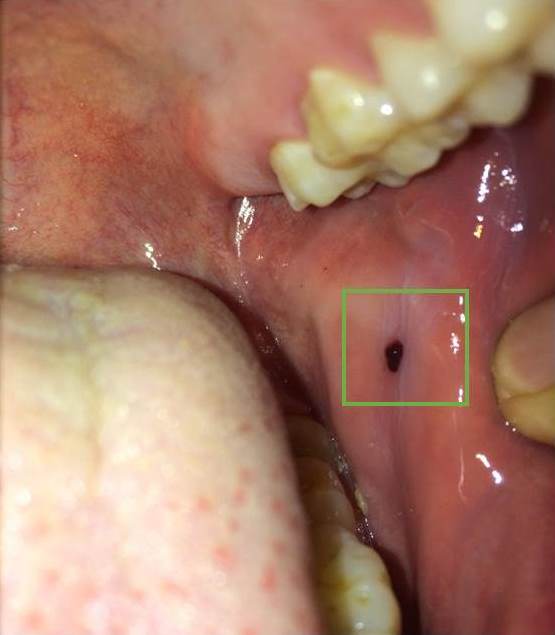


Figure S1: Hematoma image Figure S2: Oral carcinoma image


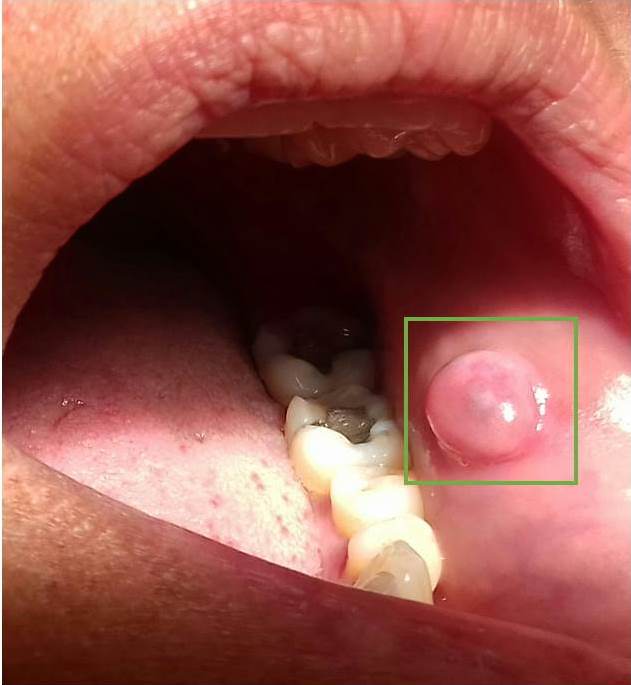


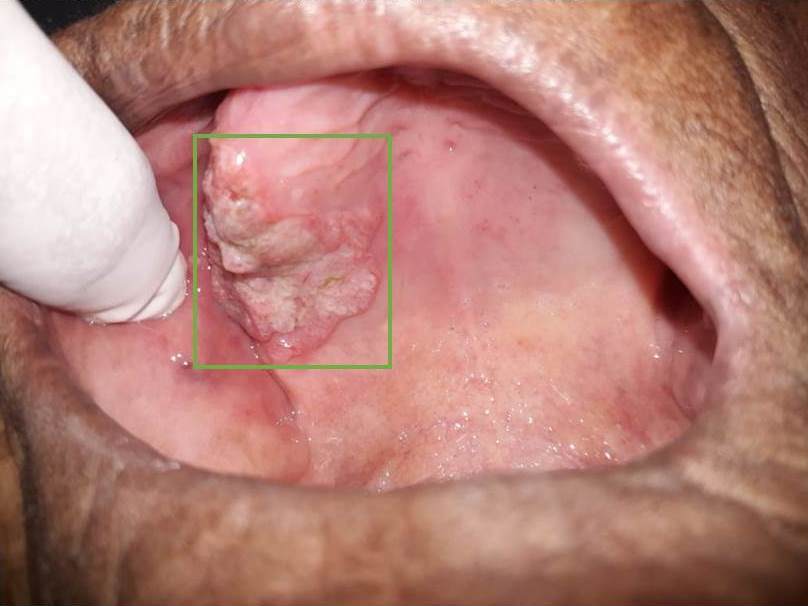


Figure S3: Verrucous carcinoma image Figure S4: Oral fibrous hyperplasia image
